# Medical and Physical *Intelligence*

**Published:** 1799-05

**Authors:** 


### MEDICAL AND PHYSICAL *INTELLIGENCE*,

### (Original and Selected.)

DR. E. A. HOLYOKE, of Salem, Maſſachuſetts, has contrived an eaſy method of preparing the *ſal aëratus*, or the carbonate of potaſs, the particular proceſs of which, on account of its ſimplicity and practical advantage, we take this opportunity of laying before our readers.

" To obtain this ſalt, in its moſt perfect form, diſſolve as much of the pure vegetable alkali, in boiling rain, or other pure water, as poſſible; filter the ſolution through paper, pour it into a jar of ſtone or earthenware, cover the veſſel in ſuch a manner that the air may have acceſs to it, but ſo as to exclude all duſt or foreign matter. Let it be hung by a cord in a fermenting vat or ciſtern for a month or two, in which time a great many cryſtals will be formed: the ſuperfluous liquor may be poured off, and the ſalt dried in what is called Hippocrates' ſleeve. This liquor may be again ſaturated with more alkali, and a ſecond time expoſed to the air in the ciſtern, without any loſs."

Although this, no doubt, is the moſt perfect mode of preparation, yet the following is much leſs troubleſome, and perhaps of equal advantage for all medicinal purpoſes, and has for the moſt part been employed by Dr. Holyoke, in preference to the former:—

" Take a large wooden box; bore eight or ten holes, half an inch in diameter, in the ſide of it, juſt below the lower edge of the cover, at nearly equal diſtances all round; bore alſo as many holes in the circular bottom of the box, cloſe to the edge of it; then take another box of the ſame kind, but of a ſmaller diameter by half or three quarters of an inch; place this in the larger box, and to keep it ſteady, thruſt three or four wooden wedges between the two boxes. The deſign of the outer box is merely to prevent any duſt or dirt from getting into the ſalt, while the holes in it ſuffer the fixed air to be freely admitted.

" The two boxes being thus prepared, fill the inner one with the pureſt ſalt of tartar, or any pure fixed vegetable alkali: put its cover on the outer box, leaving the inner one uncovered; ſling the double box, thus filled, with a cord, and ſuſpend it in a diſtiller's vat, or ciſtern, while the waſh is fermenting, a little above the liquor, or in an empty ciſtern, if it has been much uſed, and ſtill retains the fixed air: let it remain in this ſituation for ſix weeks or two months, or longer; let it then be taken out, and the ſalt, now fully ſaturated with the acid, be expoſed to the ſun and air to dry.

" The ſalt thus prepared, does neither deſloreſce nor deliqueeſce in the open air, and for all common purpoſes is, I believe, equal to that prepared by cryſtallization;—it is much more tolerable to the palate, and may be taken in larger doſes than the mere alkali; and as it is decompounded by vegetable as well as mineral acids, it may be exhibited, inſtead of the alkali, in perhaps every caſe where the latter is proper, unleſs the fixed air is judged improper.

" I commonly direct about two drachms, or rather more, of this ſalt, to be diſſolved in three ounces of pure water; a large ſpoonful of this ſolution, added to the ſame quantity of good vinegar, or lemon juice, at the inſtant of of fwallowing it, makes an agreeable dofe. But the tafte of this folution is fo mild, that if the prefcriber chufes, a fpoonful of it may be fwallowed alone firft, and as much vegetable acid immediately upon it, in which cafe none of the gas will be loft.

"When *acidity* abounds in the *firft paffages*, a little of this falt, added to any bitter infufion, or the dry falt, added to powder of columbo, or any peptic powder, is an effectual antacid.

"In *calculous cafes* this falt is recommended by writers, particularly by the celebrated Dr. CULLEN, in his '*Materia Medica*,' Vol. II. Ch. 13, as being an happy expedient, for conveying larger quantities of alkali into the ftomach, than it can bear in its natural ftate."

---

The *Economical Society of Batavia* propofe the following important queftion, and invite the fcientific inquirer, of whatever nation, to give a fatisfactory anfwer, viz.—"*Are there any means, hitherto unknown, adequate to the purpofe of purifying corrupted water fo completely, as to remove its tafte and fmell, without the admixture of any unhealthy or noxious ingredient, and rendering it a clear, refrefhing, and wholefome beverage; and what are thofe particular means?*" Six thoufand florins, or about *five hundred guineas*, are offered as a reward for a conclufive anfwer.

The following are the conditions which ought to be attended to by competitors for this handfome prize: 1. the procefs muft not be expenfive or troublefome, nor fhould it be attended with a great confumption of fuel, fo that it may be employed at fea, in fhips deeply laden, and expofed to violent motion; 2. it muft be fuch as can be eafily executed by feamen; 3. when conducted in different climates, it muft always give the fame refult; 4. it ought to produce no pernicious effects, either by corrofion or otherwife, on the coppers in which the mariners prepare their food.

One third of the prize will be due to the inventor, when, by communicating his fecret to the Committee of the Society, he fhall have enabled them to make repeated experiments, on board of fuch veffels as they fhall think proper; and the remaining two thirds, after they have made thefe experiments, on board of feveral veffels, in different climates, and fhall be convinced by fatisfactory accounts, that the means propofed have proved effectual. The memoirs on this fubject are to be directed to J. J. DESSOUT, fecretary-general to the Batavian Economical Society, at Haarlem, previous to the 28th of February, 1800.

---

In the 'Recueil periodique de la Societé de Medicine de Paris.' No. XXX. we meet with an interefting memoir, by Cit. GOLDSCHMID, *On the utility of factitious mineral waters, and the preference they deferve to thofe of the natural fprings.* The author fubmits to the fociety, the examination of the artificial waters manufactured by him, in imitation of thofe prepared by the hand of nature, in the mineral fprings of Seltz, Spa, and Sedlitz. The grounds on which he maintains the fuperiority of his artificial compounds, over the natural productions, are as follow: 1ft, The feafon has a remarkable influence on the natural fpring-waters, which in rainy feafons lofe a confiderable fhare of their virtues. 2d, It is proved by chemical analyfis, that a part of their gas and other conftituent parts efcape in tranfporting and keeping them for fome length of time; and 3d, They can only hold in folution fuch a quantity of metallic ingredients, as is proportionate to the gas and acids capable of diffolving them.

On the other hand, Cit. Goldfchmid boldly afferts, that the mineral waters of his manufacture are more efficacious, and lefs expenfive; as they not not only contain a greater portion of gas, but can also be composed of whatever quantity and quality of salt, or earth, may be judged necessary: and, as they may be prepared at the time they are wanted, they will lose no part of their virtues. Lastly, they are incomparably more economical and convenient to the purchaser, inasmuch as they are stronger, and consequently do not require to be used in such large and nauseating doses as the natural waters; a circumstance which, together with their reduced price, renders them an object worthy the attention of the consumer. In a future Number, we shall present our readers with a more detailed account of these factitious waters.

---

The following method of *preserving apples from frost*, which is used in North America, as well as in some parts of Germany, we extract from the First Number of Dr. ANDERSON's 'Recreations in Agriculture, &c. &c.' a periodical work, which we have no doubt will be found uncommonly useful to the farmer, the naturalist, and to readers in general. The apples are kept in a small apartment appropriated for that purpose, on the highest floor of the house, immediately beneath the roof, in which no fire is ever lighted, and which is therefore more exposed to the cold than any part of the building; yet it is found, by invariable experience, that if a thin linen cloth be thrown over the apples, before the frost commences, the fruit under it is never injured, however intense the frost may have been. Linen only is used for that purpose, as a covering of woollen cloth has been found ineffectual.

Dr. Anderson also communicates to us, from the Memoirs of the Royal Society of Agriculture, in Paris, the following discovery for *preventing the blossoms of fruit trees from being damaged by early Spring frosts*. If a rope, (a hempen one he presumes), be intermixed among the branches of a fruit tree in blossom, and the end of it brought down so as to terminate in a bucket of water; and should a slight frost take place during the night, in that case the tree will not be affected by the frost; but a film of ice of considerable thickness, will be formed on the surface of the bucket, in which the rope's end is immersed; although it often happened that another bucket of water, placed beside it, for the sake of experiment, had no ice at all upon it.

---

The following singular and ingenious method of multiplying the *Fritillaria Regia, Linn.* is described in the second volume of the *Collection of Memoirs and Observations on Botanical and Economical Subjects*, by the late professor *Hedwig*, of Leipzig. When this plant is in full blossom, the flowers, leaves, and upper part of the root must be cut and wrapped in several folds of writing paper, so that it be entirely covered; it is then to be moderately pressed between two boards, and in the course of a few months there will be seen several small roots germinating at the lower extremity of this apparently decayed vegetable body.

---

Another volume of the *Transactions of the Natural-History Society*, at *Copenhagen*, has been lately submitted to the public. It contains a description of several new objects belonging to the department of Natural History, together with their representations in engravings. Although the work itself is written and printed in the Danish language, the characters and descriptions of the plants are uniformly given in Latin. The whole collection now consists of four volumes.

Professor Knackstaedt, of Petersburg, who is favourably known to the medical reader, by several useful publications, has lately written an account of the medicinal properties of the *Enula Helenium*, inserted in the last volume of the *Memoirs of the Institute of Petersburg*, and in which he maintains that this plant, given internally, and at the same time applied externally, has proved a remedy of great efficacy in curing the *tinea*, *itch*, and other cutaneous diseases.

---

Dr. Mitchill, of New York, in concluding his letter to Dr. Haworth, of Oxford, on the subject of Medical Geography, some interesting parts of which we have communicated to our readers, p. 254 & seq. of this Number, makes the following curious remarks, concerning the chemico-physical tracts of *old Mayow*.

" That this man (says Dr. Mitchill,) who was a London physician, and a fellow of All-souls-college; his brilliant discoveries, and his book which contains the account of them, should all be forgotten, in his own country as well as abroad, within less than one hundred years, so effectually that *Franklin* could never have read his explanation of water spouts, nor Scheele his detection of dephlogisticated air, nor *Girtanner* his manner of accounting for muscular action, nor any body else, what he has left on respiration, and on the condition of the unborn fœtus, and unhatched chick—are among the most singular occurrences in the literary history of the eighteenth century."

---

Cit. Moricheau Beauchamp has lately presented the medical reader with a memoir, communicated to the Medical Society of Paris, and inserted in their 'Recueil Periodique,' No. xxviii. It is an account of a *particular affection of the heart*, seen in the dead body of a man, whose disorder was a complicated nasal hæmorrhage, which announced nothing extraordinary in the region of the heart. On opening the body, however, there was found a considerable quantity of blood coagulated in the stomach and the intestines; and the heart was much larger than that of a person in a state of health. At the posterior and lateral part of the left ventricle, was observed a round protuberance supported by a very large pedicle, not unlike the head of a thigh-bone attached to its neck, but of a size about a third part bigger. The heart was of a lively red colour; the pedicle somewhat darker. The pericardium covered the whole, and closely adhered to it, particularly to the convex part, which offered considerable resistance to the touch, and was of a cartilaginous hardness. On making a transverse incision, with some difficulty, a sound was perceived resembling that produced by cutting a cartilage: it exhibited a cavity of the bigness of a common nut, which penetrated the left ventricle, under the mitral valves. The interior part of this tumor presented two kinds of substances: the outermost was greyish; the other appeard not unlike the parenchyma of the heart; being soft in some parts, while in the middle of the opening could be distinguished a bony circle, large enough to admit the forefinger. Most of the valves of the heart were ossified.

---

Two eminent German booksellers have lately advertised, for sale, that magnificent edition of " *Albini Tabulæ Anatomicæ Sceleti & Musculorum*: cum Tab. Aen. Sceleti, xxxiv. & Musculorum xxvi. *Lugduni Batav.* 1747;" in two volumes, large folio. They offer this work at different prices; viz. *eight* and *nine Carolins*, or from eight to nine pounds British currency: the former reduced price is proposed by Mr. *Koel*, of Würzburg, who in the

Jena

Jena Gazette, solicits orders for this classical work. Our readers will perhaps recollect, that Professor *Soemmering*, of Mayence, who now resides at Frankfort, has lately published one of the most perfect engravings of a *female skeleton*, purposely designed as a counter-part to Albinis's male skeleton, which wanted the female; and, by the addition of which, that collection is now rendered one of the most splendid and complete anatomical works we possess.

---

There is at present in the French press an extensive work on *comparative anatomy*, from the able pen of Cit. *Cuvier*, whose talents for observation, and accuracy in description, leave no doubt that we shall be presented with a performance of superior excellence.

---

Professor GOETTLING, of Jena, endeavoured to procure an atmospheric air in the driest possible state, by means of caustic alkali. He introduced into this air, under mercury, some crystals of the most concentrated, fuming sulphuric acid, which he had obtained from Saxon vitriol. There were no longer any fumes perceptible in this exsiccated air; hence, as Mr. G. observes, it is probable, that this imperfect vitriolic acid, decomposes the water of the atmosphere, and thus becomes perfect sulphuric acid.

---

Dr. SCHERER, the chemist of Jena, now of Gotha, has ascertained by repeated experiments, that *Canton's Phosphorus* is not in the least luminous, if it be exposed to the light of the moon.

This Gentleman has also communicated to Professor Tromsdorf, the ingenious remark made by the Privy Counsellor, VON GOETHE, that putrid wood is luminous only when for some time exposed to the influence of the atmosphere. See *Tromsdorf's Journal of Pharmacy*, Vol. III. No. II.

---

By the late experiments of Dr. JAEGER *, as well as those by the Aulic Counsellor HILDEBRAND †, (who prepared azotic gas with much trouble, by the deflagration of nitre,) and the additional confirmation of Cit. *Van Mons* ‡, it is now certain that *phosporus is not luminous* in the *purest azotic gas*, as was formerly maintained by Professor Goettling. This phenomenon took place only when a small quantity of oxygen was present in the azote, and continued only during the consumption of the oxygen.

---

Mr. MARUM, the Dutch chemist, has made some very interesting experiments, on the *temperature of the electric fluid*, and found that a thermometer, placed before the conductor of Tayler's famous machine, rose in a rarefied atmosphere, from 45° to 151½°. He also succeeded in converting several solid bodies, by means of electricity, into elastic fluids.

---

Dr. SCHERER considers it very probable, that the *carbon*, produced in the well-known experiments of Dr. Pearson, and repeated by him with equal success, might have been separated from *phosphorus*, which Dr. Girtanner supposes

---

* *Gren's* Physical Journal; Vol. II. No. IV. † *Crell's* Annals of Chemistry, for 1796; Vol. I. p. 255. ‡ *Gren's* Journal; Vol. III. No. III.

fuppofes to be a compound body, and which in a procefs of combuftion, in the oxygenated muriatic acid acquires, according to Dr. Scherer, a perfectly black colour.

---

When Profeffor HELLE, placed fmoking phofphorus in pure nitrous gas, obtained from copper filings diffolved in the nitric acid, he obferved that although the *phofphorus* emitted no vapors, yet it appeared to have undergone *fome change*, as a yellow fluid fubftance was at the fame time produced. On adding a fmall quantity of oxygen, the ufual red fumes of the nitric acid were generated, but foon after this a grey coloured column of vapor defcended to the bottom; the confequence of which was, a univerfal change of colour in the folution, which prefented a highly interefting fpectacle. See *Gren's Phyfical Journal*: Vol. III. p. 93.

---

The *fulphuric acid* has been fuccefsfully *defoxygenated*, in the humid way, by means of the fulphuret of potafs. Cit. *Van Mons* made this conclufive experiment, and obferved that a folution of this falt, after ftanding for a confiderable time on pulverized charcoal, had been changed into a fulphate of potafs, (hepar fulphuris,) and difengaged fulphurated hydrogen gas. Here, therefore, the carbon had attracted the acid of the oxygen, and imparted its bafe, the fulphur, to the alkali. *Ibid.* p. 230.

---

The fame ingenious chemift fucceeded in accomplifhing a true *combination of the oxygenated muriatic acid* with ammoniac, without any decompofition of the combining fubftances having taken place in the experiment. This falt is poffeffed of the furprifing property of detonating in a requifite degree of heat, as well as in the open air, and in all fluids which do not decompofe it.

---

Profeffor KLAPROTH difcovered the *vegetable alkali* to be likewife a conftituent part of *mineral* fubftances. In the decompofition of the foffil, called *leucit*, he found from 20 to 22 parts of potafs in the hundred. The Profeffor promifes to furnifh us with the analyfis of this fubftance, in the fecond volume of his work, entitled, "*Contributions to Phyfical Knowledge*," in German.

---

Profeffor ABILGAART, in a fimilar manner, found the *potafs* completely formed as a conftituent part of *animal blood*. For, on treating the blood of horfes with the nitric acid, without the aid of fire, he obtained true nitre, by cryftallization.

---

In order to procure the *potafs chemically pure*, and free from the fulphuric and nitric acids, Profeffor WURZER points out the following procefs: Let the alkali, feparated from tartar by means of combuftion, be faturated in diftilled vinegar, and the folution afterwards be diluted with diftilled water. This clarified folution ought then to be combined, firft with the acetate of barytes, and next with the acetate of filver gradually dropped into it, as long as any precipitation takes place. After this the fluid part is to be filtered, and the folid part completely dried by evaporating the humidity; the dry falt is now to be calcined in a filver crucible, till all the vinegar be diffipated. Thus the refiduum exhibits a perfectly pure vegetable alkali, which which requires to be diffolved and clarified only in diftilled water, to render it fit for all chemical purpofes.

---

According to an account inferted in the third volume, fourth number of the late Profeffor *Gren's 'Annals of Phyfics,'* Mr. FABRONI, of Florence, is faid to have proved, that alkohol is not effentially neceffary to the formation of ether, and that he has prepared a perfect ether without the aid of vinous fpirit. On the ftrength of this fact, Mr. Fabroni propofes to eftablifh a new theory of the formation of ether, which, together with a detailed defcription of the procefs, the author has pledged himfelf to lay before the public.

---

*La Metherie* in his 'Obfervations,' Tom. xliii. p. 464, as well as *Gren* in his *Phyfical Journal*, vol iii. p. 45. have feverally obferved that the mineral or foffil alkali is completely formed in the *Salfola Soda Linn.*; a circumftance by which this plant is fufficiently diftinguifhed from all others, while it contains not the leaft particle of either potafs or calcareous earth.

---

According to a ftatement of facts contained in *Tromsdorf's Journal of Pharmacy*, vol. iii. p. 345, Mr. COPPENS has decompofed fea-falt, by mixing one part of it with four parts of lime and two parts of fand; making cakes of this mixture, and then allowing the mineral alkali to cryftallize fpontaneoufly on the furface of thefe cakes.

---

Citizen GUYTON MORVEAU has obferved, that the carbonate of barytes diffolved in water impregnated with carbonic acid gas, is the moft effectual teft, or re-agent, for difcovering the prefence of the fulphuric acid. It is further afferted, that the muriated mineral alkali is likewife readily decompofed by the application of that teft.

---

In decompofing fal ammoniac, by means of potafs, Cit. VAN MONS obtained a falt in cryftals of a regular rhomboidal figure, and a confiderable fize. According to the chemical experiments made with it, this is a triple falt, confifting of the muriatic acid which has formed a double neutral compound, partly with the vegetable alkaline falt, and partly with the ammoniac. Hence it may be underftood how it happens, that by means of the vegetable alkali, a very fmall proportion of ammoniac only can be feperated from the ammoniacal falt.

---

Chemifts, in general, have hitherto paid little attention to the *multiplied combinations of falts*. This fubject has lately received fome illuftration from the experiments of Prof. LINK: he difcovered ammoniacal, magnefian, and fulphurated potafs and foda; ferruginous and fulphurated potafs; ferruginous fulphates of copper and nickel; fulphates of zink, and ammoniacal fulphate of nickel combined with cobalt.

---

On diftilling fixteen ounces of the fulphate of filex, which had been previoufly well wafhed and beat, with four ounces of powdered carbon, in a Waldenburg retort, over a ftrong fire, Profeffor LAMPADIUS obtained only a fmall portion of fulphur, but two ounces and a half of a fluid, the fmell of which refembled hepatic gas, which was highly inflammable, of a greater fpecific gravity than water, and befides preferved its fluid ftate under water, in a temparature of 10° of de Lüc, or ° of Fahrenheit. This fluid, after being expofed for fome minutes to the frefh air, was changed into a true fulphur.

Mr.

Mr. Lampadius, on this occaſion, propoſes the queſtion : "Is ſulphur in a perfectly deſoxydated ſtate always a fluid? or was it here diſſolved in the hydrogen which became diſengaged from the latent portion (*Rückhalt*—remainder) of water in the carbon?"

Dr. Richter, the Pruſſian Chemiſt, has lately publiſhed two valuable chemical proceſſes, viz. the preparations of the *cryſtallized acid of lemons* from the juice of either freſh or putrid lemons, currants, &c, and the *citrate of iron;* but as the room allotted to this department of our Journal is already preoccupied, we are, for the preſent, obliged to defer the particulars of theſe preparations, as well as many other *new, or improved, pharmaceutical proceſſes,* which have been lately deviſed by the ableſt chemiſts in this and other countries.

In the 86th Number of the "Annales de Chemie," we find a very ingenious, but rather diffuſe, memoir of Von Humboldt, "*on the abſorption of oxygen by ſimple earths, and its influence on the cultivation of the ſoil;*" a ſubject ſo highly intereſting, that we hope to gratify our readers with an elaborate abſtract from this memoir, early in a future Number of this Journal.

Citizen Baudelocque read on the 15th of February laſt, in the Medical Society of Paris, a memoir, communicated to him by Citizen Tarbes, officer of health at Toulouſe, relative to the Cæſarean Section, and containing inquiries and reflections on ſeveral caſes in which that operation had been performed. If our limits ſhould admit of it, we propoſe to give likewiſe the ſubſtance of this inſtructive paper in a future Number of our Journal.

In the 'Grand Hoſpice d'Humanité, the operations for *lithotomy* are now invariably performed with the *lithotome caché* of Friar *Coſme.* A late German traveller, Dr. *Ayerer,* who has viſited all Italy, in which country that operation is practiſed according to the principles of Hawkins and Cheſelden, beſtows uncommon praiſe upon this method of practice of the Engliſh ſurgeons. Profeſſor *Loder,* of Jena, in the firſt Number of his 'Chirurgical Journal' gives the preference to the inſtrument uſed by Hawkins, and next to it he employs thoſe uſed by le Cat.

Dr. Bradley propoſes to deliver a ſummer-courſe of lectures on the *Theory and Practice of Medicine,* for the accomodation of ſuch gentlemen as may be prevented, by profeſſional engagements, from attending lectures during the winter.

The firſt Lecture will be delivered on Monday, the 13th of May, at the Lecture-Room, No. 21, Great Eaſtcheap, near the Monument, at ſix o'clock in the afternoon.—Terms three guineas.

As a circumſtance of medical news, we have to notice the retirement of Dr. Matthew Baillie, as a teacher of anatomy.

To this gentleman, who was his nephew, Dr. William Hunter bequeathed the uſe of his Muſeum, for a term of years ; and, in conſequence of arrangements made ſoon after Dr. Hunter's death, the anatomical lectures were carried on, ſince that time, jointly by Dr. Baillie and Mr. Cruickſhank, in the Theatre built by Dr. Hunter, in Great Windmill-ſtreet. Dr. Baillie ſoon soon established a high reputation as a teacher, for which he was admirably calculated, by having a natural clearness of mind, much improved by habits of arrangement. If he did not equal the talents of his uncle, as an accomplished orator, he possessed all that perspicuity of description, and clearness in demonstration, which are so essential for instructing others. His own view of a subject was always distinct, and he had a happy facility of rendering it so to others. By these talents he supported the reputation of the school, and gained the esteem of his pupils, who have, we understand, conveyed their sentiments of his worth, by presenting him with a handsome piece of plate.

His last lecture consisted of a demonstration of the absorbent system; at the conclusion of which he read a short but neat address to the following effect:

Gentlemen,

"I have now finished the part which belonged to me of this course of lectures,—the last which I propose to give. For some time past, my time has been so much occupied by the ordinary duties of my profession, that I have often found it difficult to give the lectures. But I have farther been induced to sacrifice the gratification arising from filling the honourable situation of a public teacher, at this time, from some private circumstances of feeling, which however I cannot very well explain in this place.

"In the course of fifteen years that I have taught anatomy, I have endeavoured faithfully to discharge the duty which I owed to the public, and have much to thank them for the liberal indulgence which I have received; having always found you, Gentlemen, more disposed to overlook my failings, than studious to search them out.

"Lectures on anatomy will continue to be given here by Mr. Cruikshank, my late partner, whose character as a teacher is well known,—and Mr. Wilson, who has for many years superintended the dissecting-room, and has given the best promise of his abilities as a teacher."

Dr. Baillie delivered this address under evident embarrasment, arising from the feeling that it was the last time of his appearing in that situation; a circumstance which, however trifling, is so connected with mortality, that few men can be wholly indifferent to it. He retired amidst the plaudits of his audience. The Theatre on this occasion was completely crouded; and he must have felt flattered, by his last lecture being attended by not only students, but by many teachers in the different departments of medicine and surgery, and by surgeons of the first eminence.

---

Dr. William Turton, author of the Medical Glossary, is about to put in the press a translation of the "*Systema Naturæ*," of Linné, from the last edition, by *Gmelin*. It will be comprised in four large octavo volumes, and will include the later discoveries of societies and naturalists. The English natural productions will be distinguished by an asterisk. Each department will be accompanied with an appropriate introduction, descriptive plates, and an explanation of the Linnean terms. The first volume, containing the Mammalia, Aves, Amphibia, and Pisces, will be ready for publication some time in the ensuing Autumn.

The great price of Gmelin's edition, and the total exclusion of those, not well acquainted with the Latin language, from consulting the works of the founder of that system, will make this publication an acceptable present to the lovers of natural history, and an useful addition to the stock of English scientific literature.

The

The day after the firft page of this Number was fent to the prefs, we received a copy of Dr. JENNER's fecond work, intitled "*Further obfervations on the variolæ vaccinæ or cow-pox.*" 4to. 64 p.p. (price 2s. 6d. *London.* Law.

Thefe obfervations contain additional evidence in favour of Dr. Jenner's original opinion, and fome ufeful cautions refpecting the manner of conducting experiments on this difeafe, of which we fhall give an account in our next.

---

The following is a correct receipt of Mr. FORSYTH's compofition for deftroying the *new infect on apple-trees,* and of which we again take notice; as in the account given of it in our laft number, p. 201, the neceffary ingredient, lime, was accidentally omitted.

R/. To one hundred gallons of *human urine,* and one bufhel of *lime,* add cow-dung to bring it to the confiftence of paint.—If the white efflorefcence-like fubftance, in which the infects are lodged, has made its appearance, it fhould be previoufly brufhed off.

---

Cit. DE CANDOLE, of Bourdeaux, has, in a late number of the 'Bulletin of the Philomatic Society,' publifhed a defcription of the *Sennebteria pinnatifida* (Lepidium didymum, Linn.). In explanation of this *nondefcript,* Cit. Tournon, of Paris, in the 'Magafin Encyclopedique' of the 1ft Germinal (March 21ft), obferves that from Candole's ftatement it would appear to be an exotic plant, but that he had found it, three years ago, behind the mill belonging to the Carthufians of Bourdeaux, near the Medoc road, in a moift foil: he further adds, that neither Linnæus, nor Murray, mention its native foil.

---

The French National Inftitute have appointed Citizen OLIVIER to the dignity of a member of the Republican School in the branch of Zoology, in the clafs of Phyfical and Mathematical Sciences. The other, lefs fortunate competitors, were Cit. *Barthez* and *Jurine.*

---

Among the branches of fcience propofed to be made the fubject of public lectures under the projected *inftitution for diffufing the knowledge, and facilitating the general introduction of ufeful mechanical inventions; and for teaching, by courfes of philofophical lectures and experiments, the application of fcience to the common purpofes of life,* are the following:

Of heat, and its application to the various purpofes of life; of the combuftion of inflammable bodies, and the relative quantities of heat producible by the different fubftances ufed as fuel; of the principles of the warmth of clothing; of the effects of heat and cold, and of hot and cold winds, on the human body, in ficknefs and in health; of the effects of breathing vitiated and confined air; of means to render dwelling-houfes comfortable and falubrious; of the methods of procuring and preferving ice in fummer, and of the beft principles of conftructing ice-houfes; of the means of preferving food in different feafons and climates; of the means of cooling liquors, without the affiftance of ice; of vegetation, and of the fpecific nature of thofe effects that are produced by manures; of the art of compofing manures, and adapting them to the different kinds of foil; of the nature of thofe changes that are produced on fubftances ufed as food in the various proceffes of cookery; of the nature of thofe changes which take place in the digeftion of food; of the chemical principles of the procefs of tanning leather, and of the objects that muft particularly be had in view, in attempts to improve that moft ufeful art; of the chemical principles of the art of making foap; of the art of bleaching; of the art of dyeing; and, in general, of all the mechanical arts, as they apply to the various branches of manufacture.

