# Errata

**Published:** 1799-06

**Authors:** 


### ERRATA.

No. III. p. 303, line 10, after the words *last lecture*, add, *on Thursday, April 11th.*

——— p. 304, l. 18, for *Sennebteria*, read *Sennebieria.*

